# A partially function-to-topic model for protein function prediction

**DOI:** 10.1186/s12864-018-5276-7

**Published:** 2018-12-31

**Authors:** Lin Liu, Lin Tang, Mingjing Tang, Wei Zhou

**Affiliations:** 1 School of Information, Yunnan Normal University, Kunming, 650500 Yunnan China; 2 Key Laboratory of Educational Informatization for Nationalities Ministry of Education, Yunnan Normal University, Kunming, 650500 Yunnan China; 3 President’s Office, Yunnan Normal University, Kunming, 650500 Yunnan China; 4 School of Software, Yunnan University, Kunming, 650091 Yunnan China

**Keywords:** Multi-label classification, Topic model, Protein function, Probability distribution

## Abstract

**Background:**

Proteins are a kind of macromolecules and the main component of a cell, and thus it is the most essential and versatile material of life. The research of protein functions is of great significance in decoding the secret of life. In recent years, researchers have introduced multi-label supervised topic model such as Labeled Latent Dirichlet Allocation (Labeled-LDA) into protein function prediction, which can obtain more accurate and explanatory prediction. However, the topic-label corresponding way of Labeled-LDA is associating each label (GO term) with a corresponding topic directly, which makes the latent topics to be completely degenerated, and ignores the differences between labels and latent topics.

**Result:**

To achieve more accurate probabilistic modeling of function label, we propose a Partially Function-to-Topic Prediction (PFTP) model for introducing the local topics subset corresponding to each function label. Meanwhile, PFTP not only supports latent topics subset within a given function label but also a background topic corresponding to a ‘fake’ function label, which represents common semantic of protein function. Related definitions and the topic modeling process of PFTP are described in this paper. In a 5-fold cross validation experiment on yeast and human datasets, PFTP significantly outperforms five widely adopted methods for protein function prediction. Meanwhile, the impact of model parameters on prediction performance and the latent topics discovered by PFTP are also discussed in this paper.

**Conclusion:**

All of the experimental results provide evidence that PFTP is effective and have potential value for predicting protein function. Based on its ability of discovering more-refined latent sub-structure of function label, we can anticipate that PFTP is a potential method to reveal a deeper biological explanation for protein functions.

## Background

Proteins are the main component of a cell, which explain the basic activity of cellular life. The research of protein functions is of great significance in elucidating the phenomena of life [1]. Although there have been amount of protein sequences in biological database in recent years [2, 3], a small percentage of these proteins have experimental function annotations because of the high cost of biochemical experiment. In comparison with biochemical experiment, computational methods predict the functional annotations of proteins by using known information, such as sequence, structure, and functional behavior, which reduce time and effort, and have become important long-standing research works in post-genomic era [4].

The earlier computational approach for predicting protein function is to utilize the protein sequence or structure similarity to transfer functional information, such as BLAST. [5]With the rapid development of computational algorithms, an increasing types of algorithms have been introduced into the studies of predicting protein function. At present, computational methods of protein function prediction can be classified as two types: classification-based approaches and graph-based approaches. In classification-based approaches, proteins are viewed as instances to be classified, and function annotations (such as Gene Ontology (GO) [6] terms) are regarded as labels. Each protein has a feature space composed by classification feature extracted from amino acid sequence, textual repositories, and so on. Based on these annotated proteins and their attribute features, we can train the classifier on training dataset and then predict function labels for unannotated proteins. For graph-based approaches, the network structure information of proteins is used to compute the distance between proteins, and then the closely related proteins are considered to have similar functional annotations [7, 8].

In classification-based approaches, since each protein is annotated with several functions, various multi-label classifiers can be adopted. Yu et.al [9] proposed a multiple kernels (ProMK) method to process multiple heterogeneous protein data sources for predicting protein functions; Fodeh et.al [10] used the binary-relevance for different classifiers to automatically assign molecular functions to genes; a new ant colony optimization algorithm is proposed in reference [11], which has applied to protein function dataset; Wang et.al [12] applied a new multi-label linear discriminant analysis approach to address protein function prediction problem; Liu et.al [4] introduced a multi-label supervised topic model called Labeled-LDA into protein function prediction, whose experimental results on yeast and human datasets demonstrated the effectiveness of Labeled-LDA on protein function prediction. This research is the first effort to apply a multi-label supervised topic model to protein function prediction. Besides, Pinoli et.al [13–15] applied two standard topic models, including latent Dirichlet allocation (LDA) and probabilistic latent semantic analysis (PLSA) [16, 17], to predict GO terms of proteins on the basic of available GO annotations.

In the topic modeling process of reference [4], each protein is viewed as a mixture of ‘topics’, where each ‘topic’ is also viewed as the mixture of amino acid blocks. In comparison with discriminative model, such as support vector machine (SVM), a multi-label supervised topic model can transform the word-level statistics of each document to its label-level distribution, and model all labels simultaneously rather than treating each label independently. Specially, topic model can provide the function label probability distribution over proteins as an output, and each function label is explained as a probability distribution over amino acid blocks. Nonetheless, in the study of Liu et.al [4], Labeled-LDA associates each label (GO term) with a corresponding topic directly, which makes the latent topics to be completely degenerated, and ignores the differences between labels and latent topics. Therefore, Labeled-LDA isn’t able to discover the topic that represents common semantic of protein functions. For interpretable text mining, Ramage et.al [18] proposed a partially labeled LDA (PLDA), which associates each label with a topic subset partitioned from global topics set. PLDA overcame the shortfalls of Labeled-LDA, and improved the precision of text classification in experimental research.

Inspired by the application of multi-label topic model in protein function prediction and PLDA model, we introduce a Partially Function-to-Topic Prediction model (called PFTP). Firstly, we describe the related definitions by contrasting text data and protein function data. Then the topic modeling process of PFTP is described in detail, including the generative process and parameter estimation of PFTP. In a 5-fold cross validation experiment on predicting protein function, PFTP significantly outperforms five algorithms compared. All of the experimental results provide evidence that PFTP is effective and have potential value for predicting protein function.

## Methods

### Related definitions and notations

**Fig. 1:**
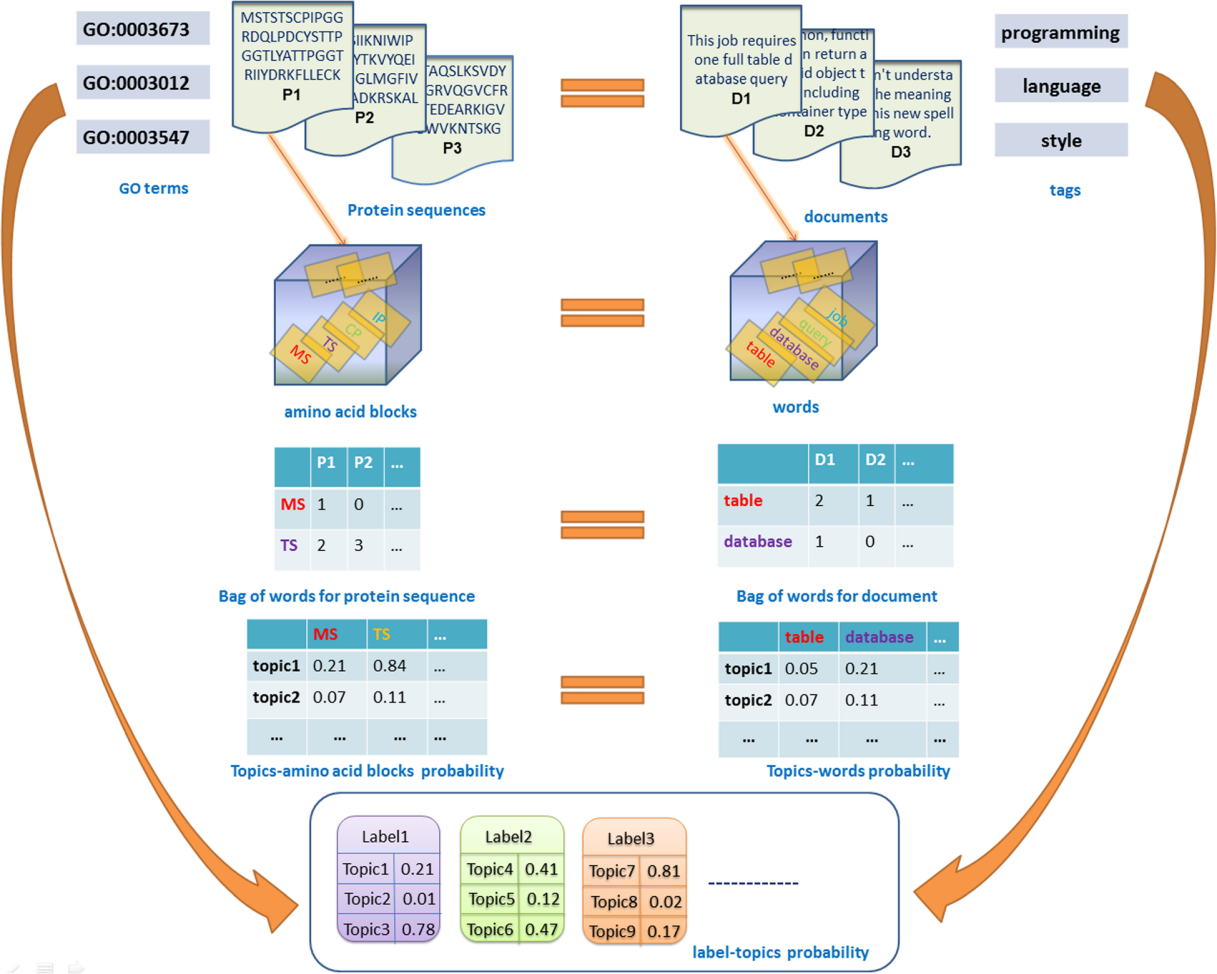
The corresponding relationship between protein function prediction and multi-label supervised topic modeling of text**.** The protein function data is shown on the left side, and the text data is shown on the right side

To better understand related objects of topic model, the corresponding relationship between protein function prediction and multi-label classification of text is first depicted in Fig. 1.

Several topic modeling concepts of protein function data and text data are displayed in Fig. 1, one on the left and the other on the right. First of all, the text dataset is composed of several documents numbered D1 to Dn, and the protein function dataset is composed of several protein sequences numbered P1 to Pn. Obviously, words are the main component of document, such as word ‘table’ and ‘database’. But for protein sequence, we consider a protein sequence to be a text string, which is defined on a fixed 20 amino acids alphabet (G,A,V,L,I,F,P, Y,S,C,M,N,Q,T,D,E,K,R,H,W). Then amino acid blocks are the main component of protein sequence, such as ‘MS’ and ‘TS’. Besides, a protein annotated by GO terms is equivalent to a document labeled by tags, so each GO term or tag can be viewed as a label, such as ‘GO0003673’ and ‘language’. According to above statements, there are three types of equivalence relations between protein function data and text data: protein sequence and document, amino acid block and word, GO term and document tag. In general, the GO term (document tag), protein sequence (document) and amino acid block (word) are observable data for dataset.

As the input for topic model, the bag of words (BoW) is constructed by computing the word-document matrix, where matrix element is obtained by counting the times of word in each document. As an instance, the word ‘table’ appears two times in document D1. Likewise for protein function data, an amino acid block - protein sequence matrix is computed for the construction of protein BoW. As an example, the amino acid block ‘MS’ appears one times in protein P1. Besides, the fixed amino acid blocks set or words set is also called ‘vocabulary’.

For topic model, a ‘topic’ is viewed as a probability distribution over a fixed vocabulary. Taking the text data as an example, the probabilities of word ‘table’ over ‘topic 1’ are 0.05. For the protein data, the probabilities of amino acid block ‘MS’ over ‘topics 1’ are 0.21. Obviously, topics are latent and needed to be inferred by topic modeling. Finally, in order to establish the connection between labels and topics, the latent topics discovered by our PFTP are divided into several non-overlapping subsets, each of which associates a label. As can be seen in Fig. 1, we split whole topic set into several groups: ‘label1’ connects with ‘topic1’ to ‘topic3’; ‘lable2’ connects with ‘topic 4’ to ‘topic 5’, and so on. It is worth noting that our PFTP define a special type of topics as background topics. The background topics are divided from whole latent topics set, and don’t associate any observable label, which express the common sematic of documents. For instance, the background topic on text dataset may be some topics with a high probability on several universal words, such as ‘text’, ‘other’ and so on. To formalize the above description, the related notations are given below.

Suppose there are *D* proteins in the protein set which compose the protein space $\mathbb{D}=\left\{1,\dots, D\right\}$, and the vocabulary of amino acid blocks is in a space of $\mathbb{W}=\left\{1,\dots, W\right\}$, then *W* is the size of vocabulary. The topic space including *K*topics is represented by $\mathbb{K}=\left\{1,\dots, K\right\}$, which is shared by whole protein set. Therefore, $\mathbb{K}$ is also called global topic space. The protein function label space is expressed as $\mathbb{L}=\left\{1,\dots, L\right\}$.

In PFTP model, the global topic space $\mathbb{K}$ is divided into *L* groups without overlap, and each group corresponds to a subspace of topic ${\mathbb{K}}_l$. Besides, there is a ‘background subspace of topics’ ${\mathbb{K}}_B$.

$$ {\displaystyle \begin{array}{l}\mathbb{K}=\left({\cup}_{l\in {\mathbb{L}}_d}{\mathbb{K}}_l\right)\cup {\mathbb{K}}_B,\kern1.5em {\mathbb{K}}_l,{\mathbb{K}}_B\subset \mathbb{K},\kern1em \\ {}\kern0.5em {\mathbb{K}}_l\ne \varnothing \kern1em \left(l\in \mathbb{L}\right),\kern1.5em {\mathbb{K}}_B\ne \varnothing, \\ {}\forall {\mathbb{K}}_i,{\mathbb{K}}_j\subset \mathbb{K},\kern1em i,j\in \mathbb{L},\kern1em i\ne j\kern1.5em \Rightarrow \\ {}{\mathbb{K}}_i\cap {\mathbb{K}}_j=\varnothing, \kern1em {\mathbb{K}}_i\cap {\mathbb{K}}_B=\varnothing \end{array}} $$

Then, each of labels is assigned a subspace of topic ${\mathbb{K}}_l$, the background topic subspace ${\mathbb{K}}_B$ associates a background label *l*_*B*_.In this case, the label space is expanded to *L* + 1 dimensions and expressed as ${\mathbb{L}}^{\prime }$. Similar to topic modeling of text in Labeled-LDA, each of topics can be represented as a multinomial distribution of parameter ${\boldsymbol{\uptheta}}_k={\left\{{\theta}_{kw}\right\}}_{w=1}^W$ (the equivalent of the topic-word matrix in Fig. 1) on the vocabulary $\mathbb{W}$, and **θ**_*k*_ obeys a Dirichlet prior distribution of hyper parameter $\boldsymbol{\uplambda} ={\left\{{\lambda}_w\right\}}_{w\in \mathbb{W}}$. But what is different about our PFTP is that each of labels *l* is represented as a multinomial distribution of parameter ${\boldsymbol{\uppi}}_l={\left\{{\pi}_{lk}\right\}}_{k\in {\mathbb{K}}_l}$ (the equivalent of the label-topics probability in Fig. 1) on its topic subspace, where *π*_*lk*_ is the probabilities of topic *k* among topic subspace ${\mathbb{K}}_l$ corresponding to label *l*. Suppose **π**_*l*_ obeys a Dirichlet prior distribution of hyper parameter **α**.

1 $$ {\boldsymbol{\uppi}}_l\sim \mathrm{Dir}\left(\boldsymbol{\upalpha} \right),\kern0.5em \boldsymbol{\upalpha} ={\left\{{\alpha}_k\right\}}_{k\in \mathbb{K}},\kern0.5em \mid \boldsymbol{\upalpha} \mid =\left|{\mathbb{K}}_l\right|={K}_l $$

We utilize a binary vector **Λ**_*d*_ to map global label space ${\mathbb{L}}^{\prime }$ to ${\mathbb{L}}_d$:

2 $$ {\displaystyle \begin{array}{l}{\mathbb{L}}_d={\left\{l{\Lambda}_{dl}\right\}}_{l=1}^{L+1}=\mathbb{L}{\boldsymbol{\Lambda}}_d\kern1em \\ {}{\boldsymbol{\Lambda}}_d={\left\{{\Lambda}_{dl}\right\}}_{l\in {\mathbb{L}}^{\prime }}=\left\{{\Lambda}_{d1},{\Lambda}_{d2},\dots, {\Lambda}_{dL},1\right\},\kern1em \\ {}{\Lambda}_{dl}=\left\{\begin{array}{l}1,\kern1em l\in {\mathbb{L}}_d\\ {}0,\kern1em l\notin {\mathbb{L}}_d\end{array}\right.\end{array}} $$

Λ_*d*, *L* + 1_ = 1 illustrates that latent background label *l*_*B*_ is assigned to each protein *d*. Then, the probabilities of ${L}_d=\left|{\mathbb{L}}_d\right|$ labels of protein *d* is represented by a weight of protein-label ${\boldsymbol{\uppsi}}_d={\left\{{\psi}_{dl}\right\}}_{l\in {\mathbb{L}}_d}={\left\{{\psi}_{dl}{\Lambda}_{dl}\right\}}_{l\in {\mathbb{L}}^{\prime }}$, and **ψ**_*d*_ obeys a Dirichlet prior distribution of hyper parameter **β**_*d*_ constrained by **β** and **Λ**_*d*_:

3 $$ {\boldsymbol{\upbeta}}_d={\left\{{\beta}_l\right\}}_{l\in {\mathbb{L}}_d}={\left\{{\beta}_l{\Lambda}_{dl}\right\}}_{l\in {\mathbb{L}}^{\prime }}={\boldsymbol{\upbeta} \boldsymbol{\Lambda}}_d,\kern0.5em \boldsymbol{\upbeta} ={\left\{{\beta}_l\right\}}_{l\in {\mathbb{L}}^{\prime }} $$

In this paper, the shared parameters of whole protein sets is called global parameter in this paper, and the parameter facing one protein is called local parameter.

### The topic modeling process of PFTP

**Fig. 2:**
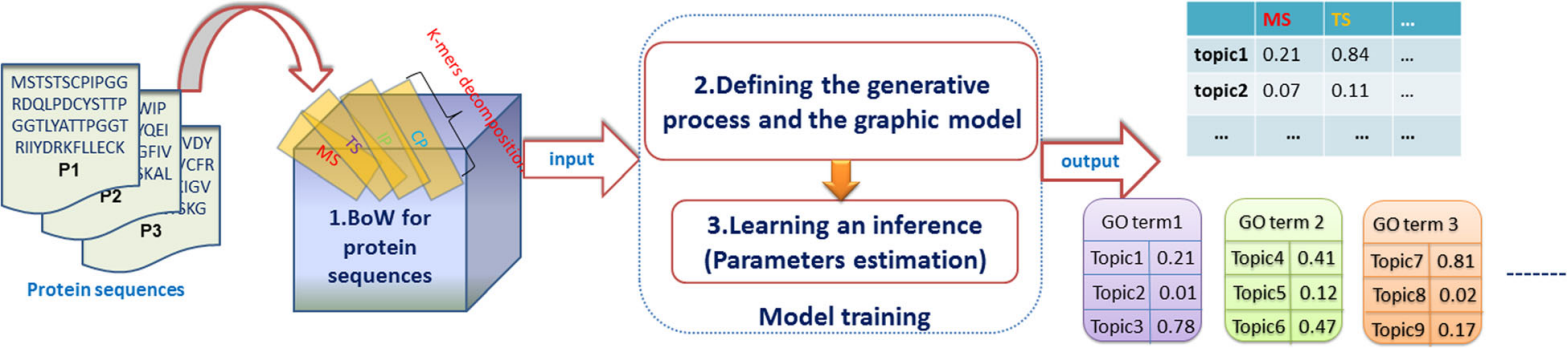
The topic modeling process of PFTP**.** The process of PFTP topic modeling is divided into three steps: BoW construction, the description of model and parameter estimation

Based on above expression, the process of PFTP topic modeling is divided into three steps: BoW construction, the description of model (the generative process or graphic model) and parameter estimation (model training and predicting).These steps are depicted in Fig. 2.

As shown in Fig. 2, PFTP model takes BoW as input. As we construct BoW of protein in exactly the same way as reference [4], this step will not repeat in this paper. There are two ways to describe our topic model, including the generative process and the graphic model. After identifying the model structure, the joint distribution of whole model is obtained. Based on this joint distribution, we can learn and infer unknown parameters of our model, which are also the output of PFTP. In fact, unknown parameters represent several matrixes. For instance, ${\boldsymbol{\uptheta}}_k={\left\{{\theta}_{kw}\right\}}_{w=1}^W$ represents the topic-word matrix in Fig. 2, and ${\boldsymbol{\uppi}}_l={\left\{{\pi}_{lk}\right\}}_{k\in {\mathbb{K}}_l}$ represents the label-topics matrix in Fig. 2.

The second and third steps are discussed in next sections. It is worth noting that the third step includes two sub-steps for realizing function prediction: model training and predicting. Both of these two sub-steps need adopt learning and inference algorithm to estimate parameters of model, and are described with more detail as follows.

#### The process of model training

PFTP takes a training protein set with known function as an input of training model. The unknown parameter includes**π**_*l*_, **θ**_*k*_ and **ψ**_*d*_. The local hidden variables include the label number and topic number of each word sample. The unknown parameter and local hidden variables can be estimated by inferring algorithm in model training.

#### The process of model predicting

For unannotated proteins, based on the estimated parameters and local hidden variables, unknown local parameter **ψ**_*d*_ and hidden variables are updating by constraining the global parameter **π**_*l*_ and **θ**_*k*_. Then, the label probabilities over protein are obtained.

### The description of PFTP model

According to the above definitions, the whole word sample *x* is composed by protein set, where ${x}_d={\left\{{\mathbf{x}}_{dn}\right\}}_{n=1}^{N_d}$. It illustrates that there are *N*_*d*_ word samples in protein *d*, **x**_*dn*_ represents one word sample. At this point, word sample **x**_*dn*_ not only associates a word number **w**_*dn*_(${\mathbf{w}}_{dn}\in \mathbb{W}$), but also is assigned a label number **l**_*dn*_(${\mathbf{l}}_{dn}\in \mathbb{L}$) and a topic number${\mathbf{z}}_{dn}\left({\mathbf{z}}_{dn}\in \mathbb{K}\right)$.

**Fig. 3:**
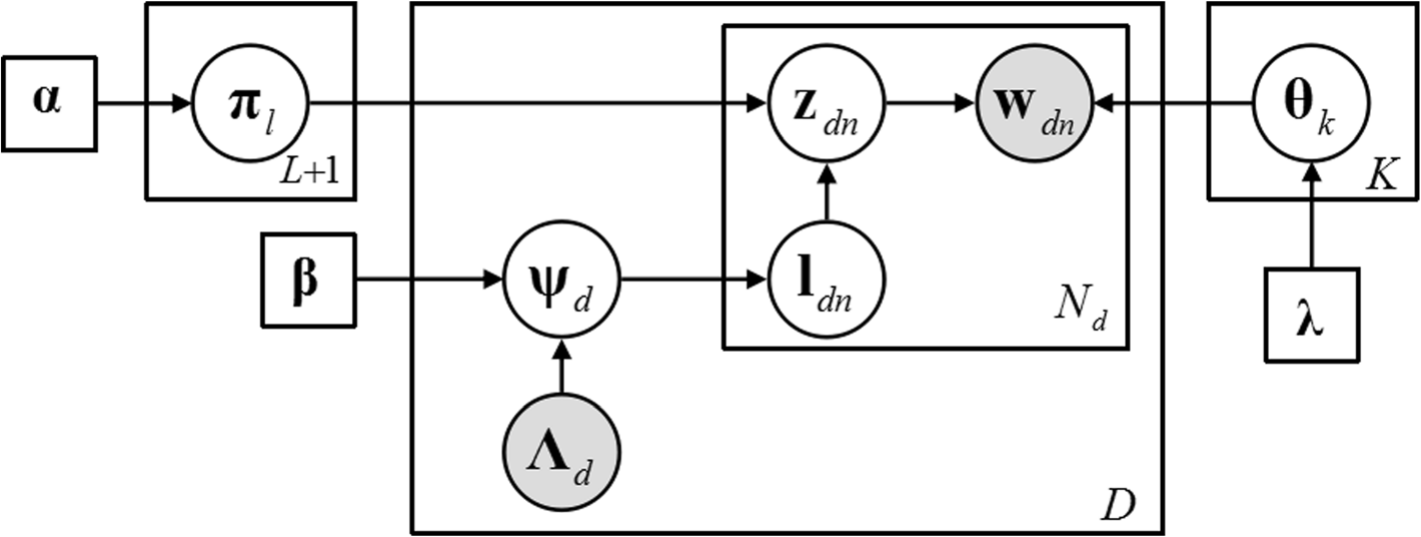
The graphic model of PTPF. Box indicates repeated contents, and the number in the bottom right corner is the times of repetition

The generative process of word sample can be described as follows. The corresponding graphical model is shown in Fig. 3.

1. For each global label $l\in {\mathbb{L}}^{\prime }=\left\{1,\dots, L,L+1\right\}$

Sample multinomial parameter vector **π**_*l*_ from *K*_*l*_ dimensions Dirichlet distribution:

4 $$ {\boldsymbol{\uppi}}_l={\left\{{\pi}_{lk}\right\}}_{k\in \mathbb{K}}\sim \mathrm{Dir}\left(\boldsymbol{\upalpha} \right),\kern0.5em \boldsymbol{\upalpha} ={\left\{{\alpha}_k\right\}}_{k\in \mathbb{K}} $$

2. For each global topic $k\in \mathbb{K}=\left\{1,\dots, K\right\}$

Sample multinomial parameter vector **θ**_*k*_ from *W* dimensions Dirichlet distribution:

5 $$ {\boldsymbol{\uptheta}}_k={\left\{{\theta}_{kw}\right\}}_{w\in \mathbb{W}}\sim \mathrm{Dir}\left(\boldsymbol{\uplambda} \right),\kern0.5em \boldsymbol{\uplambda} ={\left\{{\lambda}_w\right\}}_{w\in \mathbb{W}} $$

3. For each local protein $d\in \mathbb{D}=\left\{1,\dots, D\right\}$
  - Sample label weight vector of protein *d* from *L*_*d*_ dimensions Dirichlet distribution:

6 $$ {\boldsymbol{\uppsi}}_d={\left\{{\psi}_{dl}\right\}}_{l\in {\mathbb{L}}_d}\sim \mathrm{Dir}\left({\boldsymbol{\upbeta}}_d\right),\kern0.5em {\boldsymbol{\upbeta}}_d={\left\{{\beta}_l\right\}}_{l\in {\mathbb{L}}_d}={\left\{{\beta}_l{\Lambda}_{dl}\right\}}_{l\in {\mathbb{L}}^{\prime }}={\boldsymbol{\upbeta} \boldsymbol{\Lambda}}_d $$

where:

7 $$ \boldsymbol{\upbeta} ={\left\{{\beta}_l\right\}}_{l\in {\mathbb{L}}^{\prime }},{\boldsymbol{\Lambda}}_d={\left\{{\Lambda}_{dl}\right\}}_{l\in {\mathbb{L}}^{\prime }},{\Lambda}_{dl}=\left\{\begin{array}{l}1,\kern1em l\in {\mathbb{L}}_d\\ {}0,\kern1em l\notin {\mathbb{L}}_d\end{array}\right.,{\Lambda}_{d,L+1}\equiv 1 $$

(b) For each word sample **x**_*dn*_,
  i. Sample label number **l**_*dn*_ of **x**_*dn*_ from *L*_*d*_ dimensions multinomial distribution of parameter **ψ**_*d*_:

8 $$ {\mathbf{l}}_{dn}\sim {\boldsymbol{\uppsi}}_d\kern0.5em \mathrm{or}\kern0.5em {L}_d={\left\{{\mathbf{l}}_{dn}\right\}}_{n=1}^{N_d}\sim \mathrm{Mul}\left({\boldsymbol{\uppsi}}_d,{N}_d\right) $$

ii. Sample topic number **z**_*dn*_ of **x**_*dn*_ from *K* dimensions multinomial distribution of parameter${\boldsymbol{\uppi}}_{{\mathbf{l}}_{dn}}$:

9 $$ {\mathbf{z}}_{dn}\sim {\boldsymbol{\uppi}}_{{\mathbf{l}}_{dn}}\kern0.5em \mathrm{or}\kern0.5em {\mathrm{Z}}_d={\left\{{\mathbf{z}}_{dn}\right\}}_{n=1}^{N_d}\sim \mathrm{Mul}\left({\boldsymbol{\uppi}}_{d\;{\mathbf{l}}_{dn}},{N}_d\right) $$

iii. Sample word number **w**_*dn*_ of **x**_*dn*_ from *W* dimensions multinomial distribution of parameter ${\boldsymbol{\uptheta}}_{{\mathbf{z}}_{dn}}$:

10 $$ {\mathbf{w}}_{dn}\sim {\boldsymbol{\uptheta}}_{{\mathbf{z}}_{dn}}\kern0.5em \mathrm{or}\kern0.5em {W}_d={\left\{{\mathbf{w}}_{dn}\right\}}_{n=1}^{N_d}\sim \mathrm{Mul}\left({\boldsymbol{\uptheta}}_{Z_d},{N}_{d{\mathbf{z}}_{dn}}\right) $$

### Parameter estimation

In PFTP model, the unknown parameters to be estimated are the global label multinomial parameters $\boldsymbol{\uppi} ={\left\{{\boldsymbol{\uppi}}_l\right\}}_{l\in {\mathbb{L}}^{\prime }}={\left\{{\pi}_{lk}\right\}}_{l\in {\mathbb{L}}^{\prime },k\in {\mathbb{K}}_l}$, the global topic multinomial parameters $\boldsymbol{\uptheta} ={\left\{{\boldsymbol{\uptheta}}_k\right\}}_{k\in \mathbb{K}}={\left\{{\theta}_{kw}\right\}}_{k\in \mathbb{K},w\in \mathbb{W}}$ and the local document label weight ${\boldsymbol{\uppsi}}_d={\left\{{\psi}_{dl}\right\}}_{l\in {\mathbb{L}}_d}$; the local hidden variables are document label ${L}_d={\left\{{\mathbf{l}}_{dn}\right\}}_{n=1}^{N_d}$ and topic ${Z}_d={\left\{{\mathbf{z}}_{dn}\right\}}_{n=1}^{N_d}$; the known information are the observed label vector **Λ**_*d*_, word samples ${W}_d={\left\{{\mathbf{w}}_{dn}\right\}}_{n=1}^{N_d}$ and their joint distribution. As shown in Eq. (11):

11 $$ {\displaystyle \begin{array}{l}\kern1em p\left(\boldsymbol{\uppi}, \boldsymbol{\uptheta}, \boldsymbol{\uppsi}, L,Z,W|\boldsymbol{\Lambda}, \boldsymbol{\upalpha}, \boldsymbol{\uplambda}, \boldsymbol{\upbeta} \right)\\ {}=p\left(\boldsymbol{\uppi} |\boldsymbol{\upalpha} \right)p\left(\boldsymbol{\uptheta} |\boldsymbol{\uplambda} \right)\prod \limits_{d\in \mathbb{D}}\kern0em p\left({\boldsymbol{\uppsi}}_d|{\boldsymbol{\Lambda}}_d,{\boldsymbol{\upbeta}}_d\right)\\ {}\kern1em p\left({L}_d|{\boldsymbol{\uppsi}}_d\right)p\left({Z}_d|{L}_d,\boldsymbol{\uppi} \right)p\left({W}_d|{Z}_d,\boldsymbol{\uptheta} \right)\\ {}=\prod \limits_{l\in {\mathbb{L}}^{\prime }}\kern0em p\left({\boldsymbol{\uppi}}_l|\boldsymbol{\upalpha} \right)\prod \limits_{k\in \mathbb{K}}\kern0em p\left({\boldsymbol{\uptheta}}_k|\boldsymbol{\uplambda} \right)\prod \limits_{d\in \mathbb{D}}\kern0em p\left({\boldsymbol{\uppsi}}_d|{\boldsymbol{\Lambda}}_d,{\boldsymbol{\upbeta}}_d\right)\\ {}\kern1em \prod \limits_{n=1}^{N_d}\kern0em p\left({\mathbf{l}}_{dn}|{\boldsymbol{\uppsi}}_d\right)p\left({\mathbf{z}}_{dn}|{\boldsymbol{\uppi}}_{{\mathbf{l}}_{dn}}\right)p\left({\mathbf{w}}_{dn}|{\boldsymbol{\uptheta}}_{{\mathbf{z}}_{dn}}\right)\end{array}} $$

Based on the joint distribution, several parameter estimations can be obtained, including *p*(**π**, **θ**, **ψ**, *L*, *Z*| *W*, **Λ**, **α**, **λ**, **β**), the posterior distribution of unknown model parameters and hidden variables. In this paper, we use the Collapsed Gibbs sampling (CGS) to train a PFTP model. By marginalizing the model parameters (**π**, **θ**, **ψ**) from the joint distribution (11), the collapsed joint distribution of (*L*, *Z*, *W*) is obtained. The collapsed inference is as follows.

In the joint distribution Eq. (11), function label weight **ψ**_*d*_ only appears in *p*(**ψ**_*d*_| **Λ**_*d*_, **β**_*d*_) and *p*(*L*_*d*_| **ψ**_*d*_):

12 $$ {\displaystyle \begin{array}{c}p\left({\boldsymbol{\uppsi}}_d,{L}_d|{\boldsymbol{\Lambda}}_d,{\boldsymbol{\upbeta}}_d\right)=p\left({\boldsymbol{\uppsi}}_d|{\boldsymbol{\Lambda}}_d,{\boldsymbol{\upbeta}}_d\right)p\left({L}_d|{\boldsymbol{\uppsi}}_d\right)\\ {}=\frac{\Gamma \left({\sum}_{l\in {\mathbb{L}}^{\prime }}\kern0em {\beta}_l{\Lambda}_{dl}\right)}{\prod_{l\in {\mathbb{L}}^{\prime }}\kern0em \Gamma \left({\beta}_l{\Lambda}_{dl}\right)}\prod \limits_{l\in {\mathbb{L}}^{\prime }}\kern0em {\left({\psi}_{dl}{\Lambda}_{dl}\right)}^{\beta_l{\Lambda}_{dl}-1}\\ {}\kern0.62em \cdot \frac{\left({\sum}_{l\in {\mathbb{L}}^{\prime }}\kern0em {N}_{dl}{\Lambda}_{dl}\right)!}{\prod_{l\in {\mathbb{L}}^{\prime }}\kern0em \left({N}_{dl}{\Lambda}_{dl}\right)!}\prod \limits_{l\in {\mathbb{L}}^{\prime }}\kern0em {\left({\psi}_{dl}{\Lambda}_{dl}\right)}^{N_{dl}{\Lambda}_{dl}}\\ {}={\mathrm{C}}_1\frac{\Gamma \left({\sum}_{l\in {\mathbb{L}}^{\prime }}\kern0em {\beta}_l{\Lambda}_{dl}\right)}{\prod_{l\in {\mathbb{L}}^{\prime }}\kern0em \Gamma \left({\beta}_l{\Lambda}_{dl}\right)}\prod \limits_{l\in {\mathbb{L}}^{\prime }}\kern0em {\left({\psi}_{dl}{\Lambda}_{dl}\right)}^{\Lambda_{dl}\left({\beta}_l+{N}_{dl}\right)-1}\end{array}} $$

*N*_*dl*_ is the number of samples assigned to observed label $l\in {\mathbb{L}}_d$ of protein *d*; C_1_ is the constant of multinomial distribution coefficient:

13 $$ {\mathrm{C}}_1=\frac{\left({\sum}_{l\in {\mathbb{L}}^{\prime }}\kern0em {N}_{dl}{\Lambda}_{dl}\right)!}{\prod_{l\in {\mathbb{L}}^{\prime }}\kern0em \left({N}_{dl}{\Lambda}_{dl}\right)!}=\frac{\left({\sum}_{l\in {\mathbb{L}}^{\prime }}\kern0em {N}_{dl}\right)!}{\prod_{l\in {\mathbb{L}}_d}\kern0em {N}_{dl}!} $$

Suppose${\widehat{\beta}}_{dl}={\Lambda}_{dl}\left({\beta}_l+{N}_{dl}\right)$, ${\widehat{\psi}}_{dl}={\psi}_{dl}{\Lambda}_{dl}$. This parameter is eliminated by doing the integral of **ψ**_*d*_ in Eq. (11), the marginal distribution of local hidden variable *L*_*d*_ is shown in below:

14 $$ {\displaystyle \begin{array}{c}p\left({L}_d|{\boldsymbol{\Lambda}}_d,\boldsymbol{\upbeta} \right)={\int}_{\Psi_d}\kern0em p\left({\boldsymbol{\uppsi}}_d,{L}_d|{\boldsymbol{\Lambda}}_d,\boldsymbol{\upbeta} \right)\mathrm{d}{\boldsymbol{\uppsi}}_d\\ {}={\int}_{\Psi_d}\kern0em {\mathrm{C}}_1\frac{\Gamma \left({\sum}_{l\in {\mathbb{L}}^{\prime }}\kern0em {\beta}_l{\Lambda}_{dl}\right)}{\prod_{l\in {\mathbb{L}}^{\prime }}\kern0em \Gamma \left({\beta}_l{\Lambda}_{dl}\right)}\prod \limits_{l\in {\mathbb{L}}^{\prime }}\kern0em {\left({\psi}_{dl}{\Lambda}_{dl}\right)}^{\Lambda_{dl}\left({\beta}_l+{N}_{dl}\right)-1}\mathrm{d}{\boldsymbol{\uppsi}}_d\\ {}={\mathrm{C}}_1\frac{\Gamma \left({\sum}_{l\in {\mathbb{L}}^{\prime }}\kern0em {\beta}_l{\Lambda}_{dl}\right)}{\prod_{l\in {\mathbb{L}}^{\prime }}\kern0em \Gamma \left({\beta}_l{\Lambda}_{dl}\right)}{\left(\frac{\Gamma \left({\sum}_{l\in {\mathbb{L}}^{\prime }}\kern0em {\widehat{\beta}}_{dl}\right)}{\prod_{l\in {\mathbb{L}}^{\prime }}\kern0.1em \Gamma \left({\widehat{\beta}}_{dl}\right)}\right)}^{\hbox{-} 1}\\ {}\kern0.6em \cdot {\int}_{{\widehat{\Psi}}_d}\kern0em \frac{\Gamma \left({\sum}_{l\in {\mathbb{L}}^{\prime }}\kern0em {\widehat{\beta}}_{dl}\right)}{\prod_{l\in {\mathbb{L}}^{\prime }}\kern0.1em \Gamma \left({\widehat{\beta}}_{dl}\right)}\prod \limits_{l\in {\mathbb{L}}^{\prime }}\kern0em {{\widehat{\psi}}_{dl}}^{{\widehat{\beta}}_{dl}-1}\mathrm{d}{\widehat{\boldsymbol{\uppsi}}}_d\\ {}\propto \frac{\Gamma \left({\sum}_{l\in {\mathbb{L}}^{\prime }}\kern0em {\beta}_l{\Lambda}_{dl}\right)}{\Gamma \left({\sum}_{l\in {\mathbb{L}}^{\prime }}\kern0em {\beta}_l{\Lambda}_{dl}+{N}_d\right)}\prod \limits_{l\in {\mathbb{L}}^{\prime }}\kern0em \frac{\Gamma \left({\beta}_l{\Lambda}_{dl}+{N}_{dl}{\Lambda}_{dl}\right)}{\Gamma \left({\beta}_l{\Lambda}_{dl}\right)}\end{array}} $$

${N}_d={\sum}_{l\in {\mathbb{L}}^{\prime }}\kern0em {N}_{dl}{\Lambda}_{dl}={\sum}_{l\in {\mathbb{L}}_d}\kern0em {N}_{dl}$ is the number of observed samples of protein *d*. The integral of Eq. (14) satisfies probabilistic completeness:

15 $$ {\int}_{{\widehat{\Psi}}_d}\kern0em \frac{\Gamma \left({\sum}_{l\in {\mathbb{L}}^{\prime }}\kern0em {\widehat{\beta}}_{dl}\right)}{\prod_{l\in {\mathbb{L}}^{\prime }}\kern0.1em \Gamma \left({\widehat{\beta}}_{dl}\right)}\prod \limits_{l\in {\mathbb{L}}^{\prime }}\kern0em {{\widehat{\psi}}_{dl}}^{{\widehat{\beta}}_{dl}-1}\mathrm{d}{\widehat{\boldsymbol{\uppsi}}}_d={\int}_{{\widehat{\Psi}}_d}\kern0em p\left({\widehat{\boldsymbol{\uppsi}}}_d|{\widehat{\boldsymbol{\upbeta}}}_d\right)\mathrm{d}{\widehat{\boldsymbol{\uppsi}}}_d=1 $$

Therefore, deducing from Eq. (14), the predictive probability distribution for the label-assignment **l**_*dn*_ = *l*of sample **x**_*dn*_ is:

16 $$ p\left({\mathbf{l}}_{dn}=l|{L}_d^{\left(\backslash dn\right)},{\boldsymbol{\Lambda}}_d,\boldsymbol{\upbeta} \right)\propto \frac{\left({\beta}_l+{N}_{dl}^{\left(\backslash dn\right)}\right){\Lambda}_{dl}}{\sum_{l\in {\mathbb{L}}^{\prime }}\kern0em {\beta}_l{\Lambda}_{dl}+{N}_d^{\left(\operatorname{} dn\right)}} $$

${N}_{dl}^{\left(\backslash dn\right)}$ is the number of samples that were assigned to label *l* and word *w* in addition to the current sample *x*_*dn*_.

By the same way, in the joint distribution Eq. (11), global label parameter only appears in *p*(**π**| **α**) and *p*(*Z*_*d*_| *L*_*d*_, **π**).

17 $$ {\displaystyle \begin{array}{c}p\left(\boldsymbol{\uppi}, Z|L,\boldsymbol{\upalpha} \right)=p\left(\boldsymbol{\uppi} |\boldsymbol{\upalpha} \right)p\left(Z|L,\boldsymbol{\uppi} \right)\\ {}=p\left(\boldsymbol{\uppi} |\boldsymbol{\upalpha} \right)\prod \limits_{d\in \mathbb{D}}\kern0em p\left({Z}_d|{L}_d,\boldsymbol{\uppi} \right)\\ {}=\prod \limits_{l\in {\mathbb{L}}^{\prime }}\kern0em p\left({\boldsymbol{\uppi}}_l|\boldsymbol{\upalpha} \right)\prod \limits_{d\in \mathbb{D}}\kern0em {\prod}_{n=1}^{N_d}\kern0em p\left({\mathbf{z}}_{dn}|{\mathbf{l}}_{dn}=l,{\boldsymbol{\uppi}}_l\right)\\ {}=\prod \limits_{l\in {\mathbb{L}}^{\prime }}\kern0em \frac{\Gamma \left({\sum}_{k\in \mathbb{K}}\kern0.1em {\alpha}_k\right)}{\prod_{k\in \mathbb{K}}\kern0.1em \Gamma \left({\alpha}_k\right)}\prod \limits_{k\in \mathbb{K}}\;{\pi_{lk}}^{\alpha_k-1}\frac{\left({\sum}_{k\in \mathbb{K}}\kern0em {N}_{lk}\right)!}{\prod_{k\in \mathbb{K}}\kern0em {N}_{lk}!}\prod \limits_{k\in \mathbb{K}}\;{\pi_{lk}}^{N_{lk}}\\ {}=\prod \limits_{l\in {\mathbb{L}}^{\prime }}\;{\mathrm{C}}_2\frac{\Gamma \left({\sum}_{k\in \mathbb{K}}\kern0.1em {\alpha}_k\right)}{\prod_{k\in \mathbb{K}}\kern0.1em \Gamma \left({\alpha}_k\right)}\prod \limits_{k\in \mathbb{K}}\;{\pi_{lk}}^{\alpha_k+{N}_{lk}-1}\end{array}} $$

*N*_*lk*_ represents the number of samples assigned to topic *k* of global label *l*; C_2_ is the constant of multinomial distribution coefficient:

18 $$ {\mathrm{C}}_2=\frac{\left({\sum}_{k\in \mathbb{K}}\kern0em {N}_{lk}\right)!}{\prod_{k\in \mathbb{K}}\kern0em {N}_{lk}!} $$

Suppose ${\widehat{\alpha}}_k={\alpha}_k+{N}_{lk}$. This parameter is eliminated by doing the integral of **π** in Eq. (17), the marginal distribution of local hidden variable *Z* is shown in below:

19 $$ {\displaystyle \begin{array}{c}p\left(Z|L,\boldsymbol{\upalpha} \right)=\prod \limits_{l\in {\mathbb{L}}^{\prime }}\kern0em {\int}_{\Pi_l}\kern0em p\left({\boldsymbol{\uppi}}_l|\boldsymbol{\upalpha} \right)\prod \limits_{d\in \mathbb{D}}\kern0em {\prod}_{n=1}^{N_d}\kern0em p\left({\mathbf{z}}_{dn}|{\mathbf{l}}_{dn}=l,{\boldsymbol{\uppi}}_l\right)\mathrm{d}{\boldsymbol{\uppi}}_l\\ {}=\prod \limits_{l\in {\mathbb{L}}^{\prime }}\kern0em {\int}_{\Pi_l}\kern0em {\mathrm{C}}_2\frac{\Gamma \left({\sum}_{k\in \mathbb{K}}\kern0em {\alpha}_k\right)}{\prod_{k\in \mathbb{K}}\kern0em \Gamma \left({\alpha}_k\right)}\prod \limits_{k\in \mathbb{K}}\;{\pi_{lk}}^{\alpha_k+{N}_{lk}-1}\mathrm{d}{\boldsymbol{\uppi}}_l\\ {}=\prod \limits_{l\in {\mathbb{L}}^{\prime }}\;{\mathrm{C}}_2\frac{\Gamma \left({\sum}_{k\in \mathbb{K}}\kern0em {\alpha}_k\right)}{\prod_{k\in \mathbb{K}}\kern0em \Gamma \left({\alpha}_k\right)}{\left(\frac{\Gamma \left({\sum}_{k\in \mathbb{K}}\kern0em {\widehat{\alpha}}_k\right)}{\prod_{k\in \mathbb{K}}\kern0em \Gamma \left({\widehat{\alpha}}_{lk}\right)}\right)}^{\hbox{-} 1}\\ {}\cdot {\int}_{\Pi_l}\kern0em \frac{\Gamma \left({\sum}_{k\in \mathbb{K}}\kern0em {\widehat{\alpha}}_k\right)}{\prod_{k\in \mathbb{K}}\kern0em \Gamma \left({\widehat{\alpha}}_{lk}\right)}\prod \limits_{k\in \mathbb{K}}\;{\pi_{lk}}^{{\widehat{\alpha}}_{lk}-1}\mathrm{d}{\boldsymbol{\uppi}}_l\\ {}\propto \prod \limits_{l\in {\mathbb{L}}^{\prime }}\kern0em \frac{\Gamma \left({\sum}_{k\in \mathbb{K}}\kern0em {\alpha}_k\right)}{\Gamma \left({\sum}_{k\in \mathbb{K}}\kern0em {\alpha}_k+{N}_l\right)}\prod \limits_{k\in \mathbb{K}}\kern0em \frac{\Gamma \left({\alpha}_k+{N}_{lk}\right)}{\Gamma \left({\alpha}_k\right)}\end{array}} $$

${N}_l={\sum}_{k\in \mathbb{K}}\kern0em {N}_{lk}$ is the number of observed samples assigned to global *l* in protein set. The integral of Eq. (19) satisfies probabilistic completeness:

20 $$ {\int}_{\Pi_l}\kern0em \frac{\Gamma \left({\sum}_{k\in \mathbb{K}}\kern0em {\widehat{\alpha}}_k\right)}{\prod_{k\in \mathbb{K}}\kern0em \Gamma \left({\widehat{\alpha}}_{lk}\right)}\prod \limits_{k\in \mathbb{K}}\;{\pi_{lk}}^{{\widehat{\alpha}}_{lk}-1}\mathrm{d}{\boldsymbol{\uppi}}_l={\int}_{\Pi_l}\kern0em p\left({\boldsymbol{\uppi}}_l|{\widehat{\boldsymbol{\upalpha}}}_l\right)=1 $$

Therefore, deducing from Eq. (19), the predictive probability distribution for the topic-assignment *k* of sample **x**_*dn*_ in label *l* is:

21 $$ p\left({\mathbf{z}}_{dn}=k|{\mathbf{l}}_{dn}=l,{L}^{\left(\backslash dn\right)},{Z}^{\left(\backslash dn\right)},\boldsymbol{\upalpha} \right)\propto \frac{\alpha_k+{N}_{lk}^{\left(\backslash dn\right)}}{\sum_{k\in \mathbb{K}}\kern0.1em {\alpha}_k+{N}_l^{\left(\backslash dn\right)}} $$

${N}_{lk}^{\left(\backslash dn\right)}$ represents the number of samples that were assigned to the topic *k* of global label *l* in addition to the current sample *x*_*dn*_, ${N}_l^{\left(\backslash dn\right)}={\sum}_{k\in \mathbb{K}}\kern0em {N}_{lk}^{\left(\backslash dn\right)}$.

The integral of **θ** is same as LDA in Eq. (11):

22 $$ {\displaystyle \begin{array}{c}p\left(W|Z,\boldsymbol{\uplambda} \right)=\prod \limits_{k\in \mathbb{K}}\kern0em {\int}_{\Theta_k}\kern0em p\left({\boldsymbol{\uptheta}}_k|\boldsymbol{\uplambda} \right)\prod \limits_{d\in \mathbb{D}}\kern0em {\prod}_{n=1}^{N_d}\kern0em p\left({\mathbf{w}}_{dn}|{\mathbf{z}}_{dn}=k,{\boldsymbol{\uptheta}}_k\right)\mathrm{d}{\boldsymbol{\uptheta}}_k\\ {}\propto \prod \limits_{k\in \mathbb{K}}\kern0em {\int}_{\Theta_k}\kern0em \frac{\Gamma \left({\sum}_{w\in \mathbb{W}}\kern0.1em {\lambda}_w\right)}{\prod_{w\in \mathbb{W}}\kern0.1em \Gamma \left({\lambda}_w\right)}\prod \limits_{w\in \mathbb{W}}\kern0em {\theta}_{kw}^{\lambda_w+{N}_{kw}-1}\mathrm{d}{\boldsymbol{\uptheta}}_k\\ {}\propto \prod \limits_{k\in \mathbb{K}}\kern0em \frac{\Gamma \left({\sum}_{w\in \mathbb{W}}\kern0.1em {\lambda}_w\right)}{\Gamma \left({\sum}_{w\in \mathbb{W}}\kern0.1em {\lambda}_w+{N}_k\right)}\prod \limits_{w\in \mathbb{W}}\kern0em \frac{\Gamma \left({\lambda}_w+{N}_{kw}\right)}{\Gamma \left({\lambda}_w\right)}\end{array}} $$

Then the predictive probability distribution over the word-assignment *w*of topic *k* for observed sample **x**_*dn*_ is:

23 $$ p\left({\mathbf{w}}_{dn}=w|{\mathbf{z}}_{dn}=k,{Z}^{\left(\backslash dn\right)},{W}^{\left(\backslash dn\right)},\boldsymbol{\uplambda} \right)\propto \frac{\lambda_w+{N}_{kw}^{\left(\backslash dn\right)}}{\sum_{w\in \mathbb{W}}\kern0.1em {\lambda}_w+{N}_k^{\left(\backslash dn\right)}} $$

${N}_{kw}^{\left(\backslash dn\right)}$ is the number of samples that were assigned to the word *w* of topic *k* in addition to the current sample *x*_*dn*_, ${N}_k^{\left(\backslash dn\right)}={\sum}_{w\in \mathbb{W}}\kern0em {N}_{kw}^{\left(\backslash dn\right)}$.

Given the above, the collapsed joint distribution of (*L*, *Z*, *W*) is obtained by doing the integral of (**π**, **θ**, **ψ**) in Eqs. (14), (19) and (22).

24 $$ {\displaystyle \begin{array}{l}\kern1em p\left(L,Z,W|\boldsymbol{\Lambda}, \boldsymbol{\upbeta}, \boldsymbol{\upalpha}, \boldsymbol{\uplambda} \right)=p\left(L|\boldsymbol{\Lambda}, \boldsymbol{\upbeta} \right)p\left(Z|L,\boldsymbol{\upalpha} \right)p\left(W|Z,\boldsymbol{\uplambda} \right)\\ {}\propto \prod \limits_{d\in \mathbb{D}}\kern0em \frac{\Gamma \left({\sum}_{l\in {\mathbb{L}}^{\prime }}\kern0.1em {\beta}_l{\Lambda}_{dl}\right)}{\Gamma \left({\sum}_{l\in {\mathbb{L}}^{\prime }}\kern0.1em {\beta}_l{\Lambda}_{dl}+{N}_d\right)}\prod \limits_{l\in {\mathbb{L}}^{\prime }}\kern0em \frac{\Gamma \left({\beta}_l{\Lambda}_{dl}+{N}_{dl}{\Lambda}_{dl}\right)}{\Gamma \left({\beta}_l{\Lambda}_{dl}\right)}\\ {}\kern1.12em \cdot \prod \limits_{l\in {\mathbb{L}}^{\prime }}\kern0em \frac{\Gamma \left({\sum}_{k\in \mathbb{K}}\kern0.1em {\alpha}_k\right)}{\Gamma \left({\sum}_{k\in \mathbb{K}}\kern0.1em {\alpha}_k+{N}_l\right)}\prod \limits_{k\in \mathbb{K}}\kern0em \frac{\Gamma \left({\alpha}_k+{N}_{lk}\right)}{\Gamma \left({\alpha}_k\right)}\\ {}\kern1em \cdot \prod \limits_{k\in \mathbb{K}}\kern0em \frac{\Gamma \left({\sum}_{w\in \mathbb{W}}\kern0.2em {\lambda}_w\right)}{\Gamma \left({\sum}_{w\in \mathbb{W}}\kern0.2em {\lambda}_w+{N}_k\right)}\prod \limits_{w\in \mathbb{W}}\kern0em \frac{\Gamma \left({\lambda}_w+{N}_{kw}\right)}{\Gamma \left({\lambda}_w\right)}\end{array}} $$

To simplify computation, the Dirichlet prior distributions are symmetric Dirichlet distributions:

25 $$ {\displaystyle \begin{array}{l}\boldsymbol{\upbeta} ={\left\{{\beta}_l\right\}}_{l\in {\mathbb{L}}^{\prime }}=\left\{\underset{\left|{\mathbb{L}}^{\prime}\right|=L+1}{\underbrace{\beta, \dots, \beta }}\right\}\\ {}\boldsymbol{\upalpha} ={\left\{{\alpha}_k\right\}}_{k\in \mathbb{K}}=\left\{\underset{\mid \mathbb{K}\mid =K}{\underbrace{\alpha, \dots, \alpha }}\right\}\\ {}\boldsymbol{\uplambda} ={\left\{{\lambda}_w\right\}}_{w\in \mathbb{W}}=\left\{\underset{\mid \mathbb{W}\mid =W}{\underbrace{\lambda, \dots, \lambda }}\right\}\end{array}} $$

${\sum}_{l\in {\mathbb{L}}^{\prime }}\kern0em {\beta}_l{\Lambda}_{dl}={\sum}_{l\in {\mathbb{L}}_d}\kern0em {\beta}_l=\beta {L}_d$, ${\sum}_{k\in \mathbb{K}}\kern0.1em {\alpha}_k=\alpha K$ and ${\sum}_{w\in \mathbb{W}}\kern0.1em {\lambda}_w=\lambda W$ can be substituted to Eq. (24):

26 $$ {\displaystyle \begin{array}{l}\kern1em p\left(L,Z,W|\boldsymbol{\Lambda}, \boldsymbol{\upbeta}, \boldsymbol{\upalpha}, \boldsymbol{\uplambda} \right)=p\left(L|\boldsymbol{\Lambda}, \boldsymbol{\upbeta} \right)p\left(Z|L,\boldsymbol{\upalpha} \right)p\left(W|Z,\boldsymbol{\uplambda} \right)\\ {}\kern10em \propto \prod \limits_{d\in \mathbb{D}}\kern0em \frac{\Gamma \left(\beta {L}_d\right)}{\Gamma \left(\beta {L}_d+{N}_d\right)}\prod \limits_{l\in {\mathbb{L}}_d}\kern0em \frac{\Gamma \left(\beta +{N}_{dl}\right)}{\Gamma \left(\beta \right)}\\ {}\kern10.5em \cdot \prod \limits_{l\in {\mathbb{L}}^{\prime }}\kern0em \frac{\Gamma \left(\alpha K\right)}{\Gamma \left(\alpha K+{N}_l\right)}\prod \limits_{k\in \mathbb{K}}\kern0em \frac{\Gamma \left(\alpha +{N}_{lk}\right)}{\Gamma \left(\alpha \right)}\\ {}\kern10em \cdot \prod \limits_{k\in \mathbb{K}}\kern0em \frac{\Gamma \left(\lambda W\right)}{\Gamma \left(\lambda W+{N}_k\right)}\prod \limits_{w\in \mathbb{W}}\kern0em \frac{\Gamma \left(\lambda +{N}_{kw}\right)}{\Gamma \left(\lambda \right)}\end{array}} $$

Then, the prediction probability distribution of hidden variable **z**_*dn*_ and **l**_*dn*_can be computed from that collapsed joint distribution as a transition probability of state space in the Markov chain. Through Gibbs Sampling iteration, Markov chain converges to the target stationary distribution after the burn-in time. Finally, collecting sufficient statistic samples from the converged Markov chain state space and averaging among the samples, we can get a posteriori estimates of corresponding parameters.

Deducing from Eqs. (16), (21) and (23), the predictive probability distribution for the word-assignment *w*of topic *k* in label *l* for sample **x**_*dn*_ is:

27 $$ {\displaystyle \begin{array}{l}\kern1em p\left({\mathbf{l}}_{dn}=l,{\mathbf{z}}_{dn}=k,{\mathbf{x}}_{dn}=w|{L}^{\left(\backslash dn\right)},{Z}^{\left(\backslash dn\right)},{W}^{\left(\backslash dn\right)},{\boldsymbol{\Lambda}}_d,\boldsymbol{\upbeta}, \boldsymbol{\upalpha}, \boldsymbol{\uplambda} \right)\\ {}\propto p\left({\mathbf{l}}_{dn}=l|{L}_d^{\left(\backslash dn\right)},{\boldsymbol{\Lambda}}_d,\boldsymbol{\upbeta} \right)\cdot p\left({\mathbf{z}}_{dn}=k|{\mathbf{l}}_{dn}=l,{L}^{\left(\backslash dn\right)},{Z}^{\left(\backslash dn\right)},\boldsymbol{\upalpha} \right)\cdot \\ {}\kern1em p\left({\mathbf{w}}_{dn}=w|{\mathbf{z}}_{dn}=k,{Z}^{\left(\backslash dn\right)},{W}^{\left(\backslash dn\right)},\boldsymbol{\uplambda} \right)\\ {}\propto \frac{\left({\beta}_l+{N}_{dl}^{\left(\backslash dn\right)}\right){\Lambda}_{dl}}{\sum_{l\in {\mathbb{L}}^{\prime }}\kern0em {\beta}_l{\Lambda}_{dl}+{N}_d^{\left(\operatorname{} dn\right)}}\cdot \frac{\alpha_k+{N}_{lk}^{\left(\backslash dn\right)}}{\sum_{k\in \mathbb{K}}\kern0.1em {\alpha}_k+{N}_l^{\left(\backslash dn\right)}}\cdot \frac{\lambda_w+{N}_{kw}^{\left(\backslash dn\right)}}{\sum_{w\in \mathbb{W}}\kern0.1em {\lambda}_w+{N}_k^{\left(\backslash dn\right)}}\end{array}} $$

## Results

### Dataset

To investigate the performance of the proposed method, we utilize two types of datasets. The first one is S.cerevisiae dataset (S.C) proposed in [19], and the second one is human dataset constructed by ourselves.

In S.C dataset, there are several sub datasets that constructed from different characteristics of yeast genome. Meanwhile, each sub dataset use two kinds of function annotation standard, FunCat and GO. We mainly use the sub dataset that depends on the amino acid sequence of protein and GO. What’s more, to compare the performance of PFTP between difference label numbers, we construct a dataset named S.C-CC from S.C, which only includes GO terms belonging to cellular component. Then, there are two datasets constructed from S.C.

The human dataset is constructed from the Universal Protein Resource (UniProt) databank [2] and constructed by the similar way of reference [4]. Meanwhile, we construct two Human datasets for different word length, where the max word length of Human1 dataset is two alphabet, and which of Human2 dataset is three alphabet.

Due to the large number of GO terms in protein function dataset, we adopted a label space dimension reduction (LSDR) method to overcome the classification difficulty of classifiers. Boolean Matrix Decomposition (BMD) has been studied for LSDR recently, which can recovery the label space after classification conveniently. Therefore, a BMD method proposed in reference [20] has conducted in S.C and Human dataset. The statistics of above two datasets is displayed in Table 1. ‘*L*’ represents the number of GO terms after BMD; ‘*D*’ denotes the number of proteins in each dataset; ‘*W*’ denotes the size of vocabulary.

**Table 1:** The statistic of four datasets

Dataset	*D*	*W*	*L*
Human 1	4962	5297	1477
Human 2		400	
S.C	1692	400	1538
S.C-CC			319

### Parameter settings

PFTP model involves three parameters: *α*, *λ* and *K*. *α* and *λ* are the parameters of two Dirichlet distribution, where the larger the value of *λ*, the more balanced the probabilistic of word in a topic. According to the experience, we set *α* = 50/*K*,*λ* = 200/*W*. The settings and impact of *K* value are explained later.

In the Gibbs sampling process of model training, we set the number of Markov chain as 1, the maximum number of iterations as 2000 times, where the number of iteration of burn-in time is set to 1000. We record the state space at intervals of 50 times on converged Markov chain, and 20 times of record is conducted. In the process of model predicting, we set the number of iterations as 1000 times. After 500 times of iterations for burn-in time, we record the state space at intervals of 50 times.

### Evaluation criterias

In all of our experiments, we use three representative multi-label learning evaluation criteria, including Hamming loss(HL), Average precision(AP) and One Error. Besides, we also use three kinds of area under Precision-Recall curve proposed in reference [19], including $\overline{AUPRC}$, $AU\left(\overline{PRC}\right)$ and $\overline{AUPRCw}$. Meanwhile, the 5-fold cross validation is adopted to assess the performance of PFTP and contrast methods. The average results of 5 independent rounds are reported in following sections.

### The impact of topic number on experimental results

**Fig. 4:**
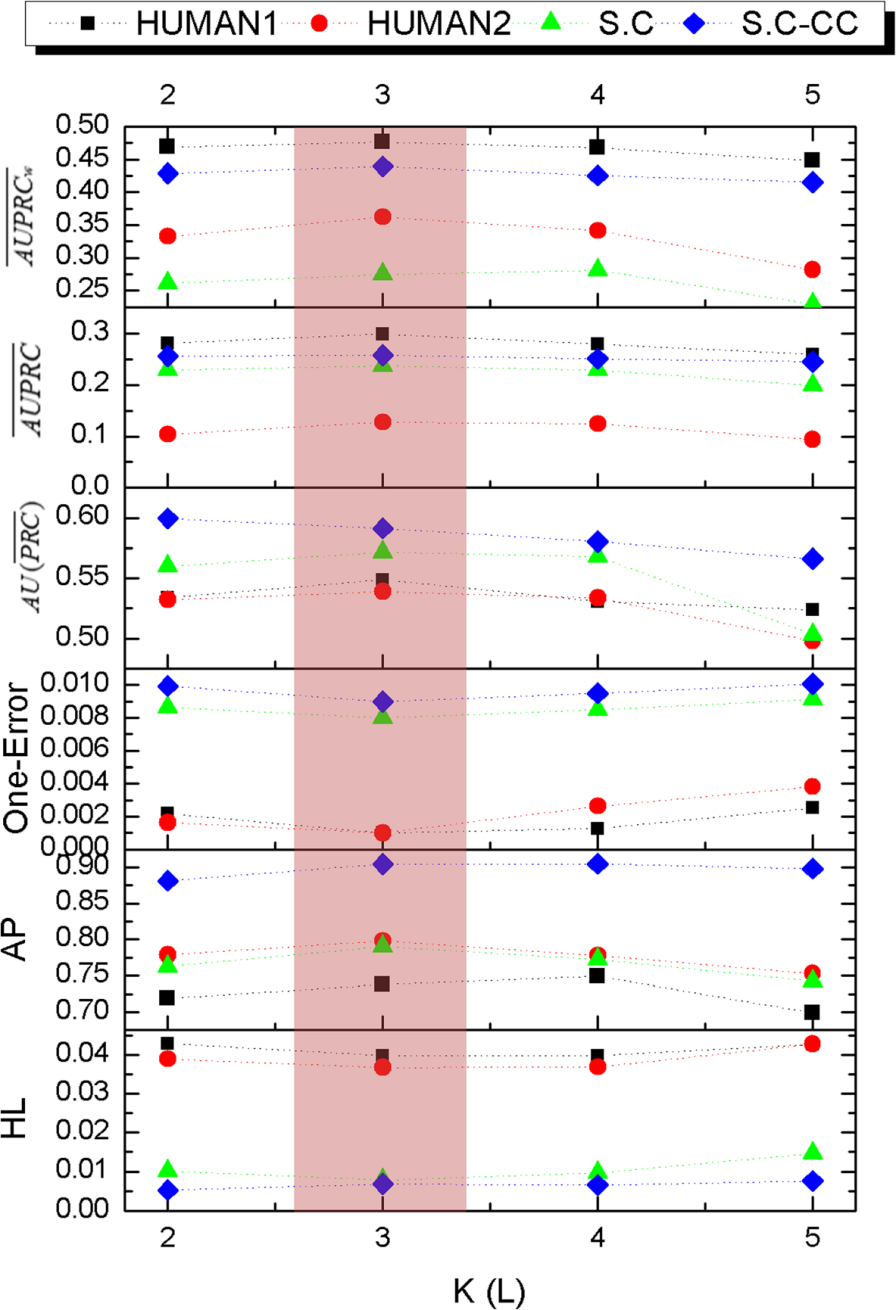
The performance comparison of different K setting. For AP, $AU\left(\overline{PRC}\right)$, $\overline{AUPRC}$ and $\overline{AUPRCw}$, the larger the value, the better the performance; for HL and One-Error, the smaller the value, the better the performance; The red background represents the best value range

*K* denotes the number of global topics. The analysis about impact of *K* on model performance is discussed in this section. According to the description of Section 2, as PFTP allocates one or more latent topics to each GO term, then the value of *K* should range from *L*to infinity in theory. Specifically, if we allocate only one topic to each GO term (*K* = *L*), then the model reduces to Labeled-LDA. Obviously, setting *K* < *L*makes our PFTP have no ability to discover the sub-structure of function. In our experiment, each function is assigned exactly the same number of topics for the simplicity of computation. For example, we set *K* = 3*L*, then each GO term corresponds to a topic set with three topics. In view of above reason, the lower bounded of *K* value is set to 2*L*. On the other hand, although theory insists that the larger *K* value equals to the more refined sub-structure of label, incorporating more latent topics per function will increase the computational load. In reference [18], the impact of *K* value on the effectiveness of PLDA model has been discussed in several texts collections. Along with the growth of topic size, the performance of PLDA model approaches a fixed value which was obtained by a non-parametric model. In other words, the infinitely larger size of topics doesn’t equal to an infinitely greater performance, but an unbearable running time. Therefore, we set the upper bound of *K* value as 5*L*based on our empirical experience and the acceptable level of time overhead. In sum, the *K*value should be set to an integer between 2*L* and5*L*. Then, the performance of PFTP under different *K*value is shown in Fig. 4.

As shown in Fig. 4, all of the evaluation criteria value is relatively stable when *K*is set to2*L*~4*L*. Nonetheless, when *K*value is greater than 4*L*, the values of AP,$\overline{AUPRC}$,$AU\left(\overline{PRC}\right)$ and $\overline{AUPRCw}$ decrease with the increase of *K*, the value of Hamming loss and One Error slowly increase with the increase of *K*. These results suggest that the optimum value range of *K* is 2*L* to4*L*. This was due to that the lower *K* value makes the fewer topics allocated to each label, and the higher *K* value makes the small difference of word distribution between topics. What’s more, the problem of huge labels is particularly obvious in protein function dataset, even if a BMD method has applied to reduce the label dimension. Therefore, we set *K* as 3*L* in our experiment.

### Evaluation against widely adopted method

**Fig. 5:**
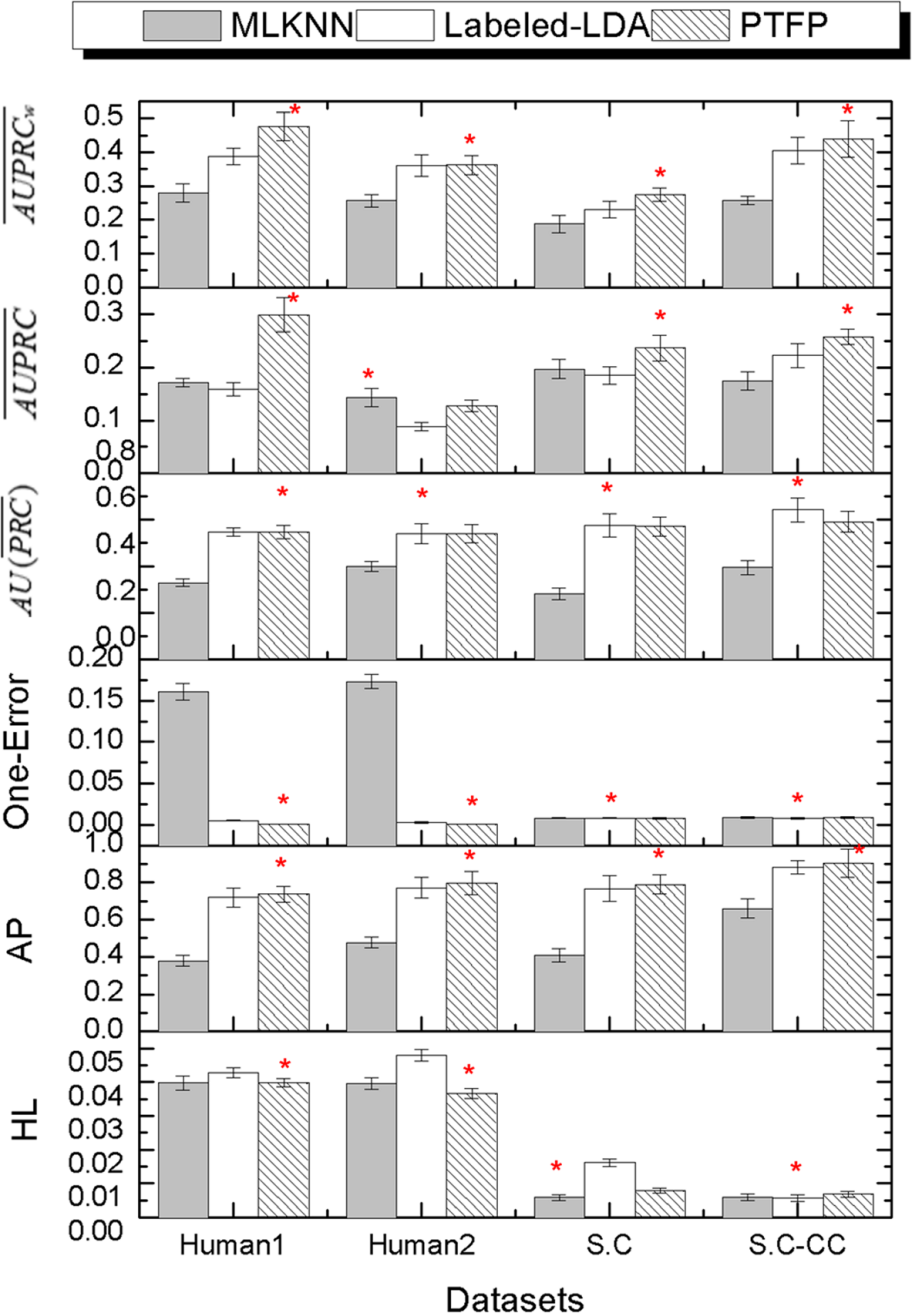
The comparison results with PTFP and Labeled-LDA. For AP, $\overline{AUPRC}$, $AU\left(\overline{PRC}\right)$ and $\overline{AUPRCw}$, the larger the value, the better the performance; for HL and One-Error, the smaller the value, the better the performance; the red asterisk on bar represents the best result on each dataset

Firstly, we compare PFTP with Labeled-LDA [4] and multi-label K-nearest neighbor (MLKNN) [21] on four datasets. MLKNN is a representative multi-label classifier and can be applied by an open source tool called Mulan [22]. Figure 5 shows the HL, AP, One Error, $AU\left(\overline{PRC}\right)$, $\overline{AUPRC}$ and $\overline{AUPRCw}$ values of these three models in SC, SC-CC, Human1 and Human2 dataset, respectively. For AP, $AU\left(\overline{PRC}\right)$, $\overline{AUPRC}$ and $\overline{AUPRCw}$, the larger the value, the better the performance. Conversely, for HL and One-Error, the smaller the value, the better the performance. The red asterisk of Fig. 4 represents the best result on each dataset.

As shown in Fig. 5, we can observe that PTPF shown more advantages in contrast to Labeled-LDA and MLKNN in four datasets. Concrete analysis is as follows:

For Human1 dataset, PFTP obtain a better performance in all evaluation criteria. On HL, PTPF achieves 9.7 and 2% improvements over Labeled-LDA and MLKNN. On One-Error, PTPF achieves 80 and 99% improvements over Labeled-LDA and MLKNN. On AP, $AU\left(\overline{PRC}\right)$, $\overline{AUPRC}$ and $\overline{AUPRCw}$, PFTP achieves 2.5, 0.2, 47 and 18% improvements over Labeled-LDA, and achieves 48, 40, 43 and 41% improvements over MLKNN. Obviously, the improvements on $\overline{AUPRC}$ and $\overline{AUPRCw}$ is more significant than $AU\left(\overline{PRC}\right)$.

For Human2 dataset, PFTP obtain a better performance in four evaluation criteria except $AU\left(\overline{PRC}\right)$ and $\overline{AUPRC}$. On HL, PTPF achieves 30 and 7.9% improvements over Labeled-LDA and MLKNN. On One-Error, PTPF achieves 66 and 99% improvements over Labeled-LDA and MLKNN. On AP and $\overline{AUPRCw}$, PFTP achieves 3.3 and 0.2% improvements over Labeled-LDA, and achieves 40 and 29% improvements over MLKNN. Nevertheless, on $AU\left(\overline{PRC}\right)$ and $\overline{AUPRC}$, MLKNN and Labeled-LDA get better results respectively.

For S.C dataset, PFTP obtain a better performance in four evaluation criteria except HL and One-Error. On AP, $\overline{AUPRC}$ and $\overline{AUPRCw}$, PTPF achieves 2.8%, 22 and 16% improvements over Labeled-LDA, and achieves 48, 17 and 32% improvements over MLKNN; on $AU\left(\overline{PRC}\right)$, the results of Labeled-LDA and PFTP are almost the same. Nevertheless, on HL, MLKNN gets better results than PFTP; on One-Error, almost identical results were obtained by these three methods.

For S.C-CC dataset, PFTP obtain a better performance on AP, $\overline{AUPRC}$ and $\overline{AUPRCw}$. On AP, PTPF achieves 2.6 and 27% improvements over Labeled-LDA and MLKNN. On $\overline{AUPRC}$, PTPF achieves 14 and 32% improvements over Labeled-LDA and MLKNN. On $\overline{AUPRCw}$, PTPF achieves 7.8 and 41% improvements over Labeled-LDA and MLKNN.

**Fig. 6:**
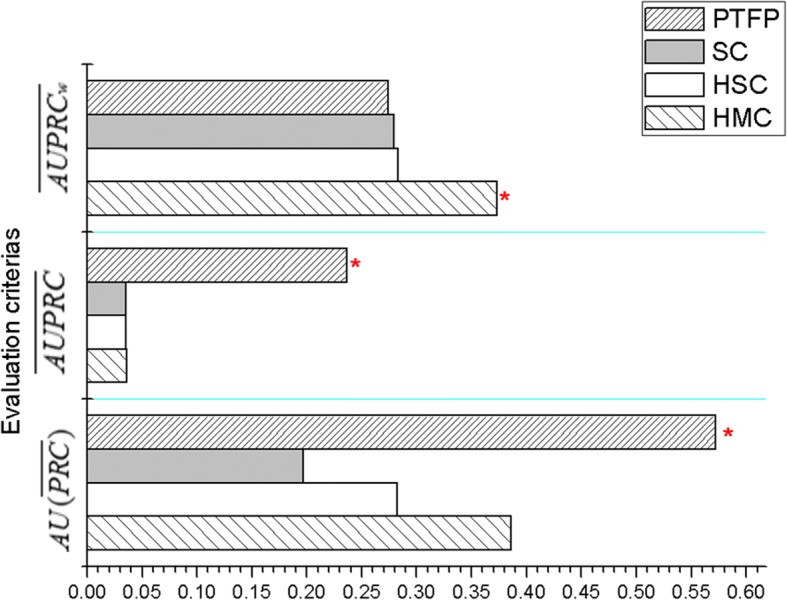
The comparison results with PTFP and HMC/SC/HSC. For three evaluation criteria, the larger the value, the better the performance, and the red asterisk on bar represents the best result on each dataset

Besides, we compare PFTP with three hierarchal multi-label classification (HMC) algorithm based on decision tree, namely HMC/SC (single-label classification)/HSC (hierarchical single-label classification) [19]. These three algorithms have been studied on protein function prediction dataset and proved to be a kind of multi-label classifiers with great performance. Since the results of CLUS-HMC/SC/HSC in reference [19] are only on S.C dataset, the comparison results with our PFTP are also on S.C dataset, and are plotted in Fig. 6.

On $\overline{AUPRC}$, our method exhibits dominant advantage against all of the three comparison methods. The performance improvements are 85, 85 and 84% against CLUS-SC, CLUS-HSC and CLUS-HMC, respectively. On $AU\left(\overline{PRC}\right)$, PTPF achieves 65, 51 and 32% improvements over CLUS-SC, CLUS-HSC and CLUS-HMC. Nonetheless, on $\overline{AUPRCw}$, CLUS-HMC gets better results than PFTP.

### The topics discovered by PFTP

The greatest strength of our protein function topic modeling is that, it can not only provide the function label probability distribution over proteins as an output, but also each function label can be explained as a probability distribution over topic subset, where each topic is represented as the probability distribution over amino acid blocks. To better understand this topic modeling process, we take GO term ‘GO0016020’ as an example, whose corresponding topics are shown in Table 2.

**Table 2:** The topics discovered by two models

Method	Topic number	words
Labeled-LDA	288	GM IH LH VH LK IG GC IC AK VM FG AM LW IK VG VW FC IG FH GK
PFTP	863	LM SM FG FC VG SG FT VM IT IM AK LG LW LK SC FK ST AG VK GM
	864	GK IC VH GV SM TH IH VM AW GM AV GE VK AG IK LV GC GL TK LK
	865	LT GC AH IK IH LH SK SW LC YM VH TG IG LG AX FW FK SF YX AM
	1	LC AC AM VW VC GM AH AV AW VH GW AK AT GC TC GH LH LW EC TH

As shown in Table 2, the 2-mers BoW is used in this example. For Labeled-LDA, the one-to-one correspondence between label and word is the key design consideration. Therefore, ‘GO0016020’ only corresponds with a topic numbered 288, and also corresponds with a probability distribution over word. The top 20 words are listed from large to small order.

For PFTP model, each GO term is a partition of global topics set. Such as for S.C-CC dataset, the number of function label is 319, while the number of global topics is three times that of the labels, that’s a total of 958(including a background topic). Therefore, each GO term corresponds with four topics (including three local topics and one background topic). The topic number 863,864,865 and 1 are the four topics corresponded by ‘GO0016020’, where the number 1 is a background topic. Likewise, the top 20 words of these four topics are listed from large to small order.

## Discussions

The results in Figs. 5 and 6 indicate that PFTP has the significant advantage against several widely adopted multi-label classifiers.

Compared with traditional multi-label classifiers(non-topic model), our method can further improve the accuracy of protein function prediction by introducing topics subset into supervised topic model, which can discover the topic that represents common semantic of documents and reflect the differences between labels and latent topics. Especially for CLUS-HMC/SC/HSC, our method exhibit the dominant advantage on $\overline{AUPRC}$. We attribute this success of our method to its utilization of BMD method on dataset. As the computation of $\overline{AUPRC}$ doesn’t bias toward the accuracy of function label annotating more proteins, and focus on the average of whole accuracy. The GO term annotating fewer proteins will be deleted after BMD processing, and recovered after predicting, but the prediction accuracy don’t reduce. In other words, the combination of PFTP and BMD can improve the average accuracy of protein function prediction.

Compared with Labeled-LDA, PFTP is able to discovery more-refined latent sub-structure of function label than Labeled-LDA. By introducing topic subset for each label in PTPF, the relationship between functions and variety words, labels and topics were disclosed. Therefore, we can anticipate that PFTP is a potential method to reveal a deeper biological explanation for protein functions.

Meanwhile, the performance comparison of different dataset is also shown in Fig. 4. For S.C-CC dataset, six evaluation criteria values vary relatively smoothly. It may be due to the fewer labels of S.C-CC dataset, then changing the *K* value doesn’t lead to great impact on prediction effect. In the comparison of S.C and S.C-CC dataset, we find that the value of AP, $AU\overline{(PRC)}$, $\overline{AUPRC}$ and $\overline{AUPRCw}$ on S.C is lower than S.C-CC, and the value of One-Error and HL is almost equal between S.C and S.C-CC. This is due to the same word space and different label number between these two dataset. The fewer labels of S.C-CC can make a higher classifying performance. In the comparison of Human1 and Human2 dataset, we find that the value of $\overline{AUPRC}$ and $\overline{AUPRCw}$ on Human1 is higher than Human2; the value of AP on Human1 is lower than Human2; the value of One-Error, HL and $AU\overline{(PRC)}$ is almost equal on Human1 and Human2. These results show that, the classification performance of PFTP on Human1 and Human2 is almost the same, which reveal that the larger word space might not obtain a better classifying performance.

## Conclusions

In this paper, we introduced an improved multi-label supervised topic model for predicting protein function. In our previous study, a multi-label supervised topic model Labeled-LDA has been applied to protein function prediction, which associates each label (GO term) with a corresponding topic directly. This way makes the latent topics to be completely degenerated, and ignores the differences between labels and latent topics. To address the faultiness, we proposed a Partially Function-to-Topic Prediction model for introducing the local topic subset corresponding to each function label. PFTP not only supports latent topics subsets within given function labels but also a background topic corresponding to a ‘fake’ function label. In a 5-fold cross validation experiment on predicting protein function, PFTP significantly outperforms compared methods. Due to the more-refined way of function label modeling, PFTP shows the effectiveness and potential value in predicting protein function through experimental studies. Meanwhile, there are several problems in topic modeling of protein function prediction to be improved, such as the introduction of protein extra features and hierarchical function label structure. However, multi-label topic model is a potential method in many applications of bioinformatics.

## Abbreviations

BMD: Boolean Matrix Decomposition
BoW: Bag of Words
CGS: Collapsed Gibbs sampling
GO: Gene Ontology
HL: Hamming loss, AP: Average precision
HMC: Hierarchal Multi-label Classification
HSC: Hierarchical Single-label Classification
LDA: Latent Dirichlet Allocation
LSDR: Label Space Dimension Reduction
MLKNN: Multi-label K-nearest neighbor
PFTP: Partially Function-to-Topic Prediction
PLDA: Partially Labeled LDA
PLSA: Probabilistic Latent Semantic Analysis
S.C: S.cerevisiae
SC: Single-label Classification
SVM: Support Vector Machine
UniProt: Universal Protein Resource
